# Patients with infusion-related reactions on fixed-dose rituximab treatment have higher body surface area than those without infusion-related reactions in adults with frequently relapsing minimal change nephrotic syndrome: a retrospective study

**DOI:** 10.1186/s40780-024-00334-0

**Published:** 2024-02-22

**Authors:** Hironobu Nishiura, Masaya Takahashi, Katsuhito Mori, Takashi Sugimoto, Masanori Emoto, Yasutaka Nakamura

**Affiliations:** 1 Department of Pharmacy, Osaka Metropolitan University Hospital, 1-5-7 Asahimachi, Abeno- ku, 545–8586 Osaka, Japan; 2 Department of Infection Control, Osaka Metropolitan University Hospital, 1-5-7 Asahimachi, Abeno-ku, 545–8586 Osaka, Japan; 3 Department of Quality and Safety Management, Osaka Metropolitan University Hospital, 1-5-7 Asahimachi, Abeno-ku, 545–8586 Osaka, Japan; 4 Department of Nephrology, Osaka Metropolitan University Graduate School of Medicine, 1-4-3, Asahimachi, Abeno-ku , 545-8585 Osaka, Japan

**Keywords:** Fixed-dose rituximab, Infusion-related reaction, Nephrotic syndrome, Body surface area

## Abstract

**Background:**

Infusion-related reactions (IRRs) are major side effects of rituximab administration. Male sex, high body weight, body surface area (BSA), and body mass index are predictive markers of rituximab-induced IRRs. However, as rituximab was not administered at a fixed dosage in a previous study, whether a higher dosage or factors associated with a larger physique are more strongly associated with rituximab-induced IRRs is unknown.

**Main body:**

Thirteen adults with frequently relapsing minimal change nephrotic syndrome (MCNS) who received an initial rituximab dose of 500 mg between September 2015 and November 2022 were retrospectively evaluated. Data on IRRs were collected from medical records. The incidence of rituximab-induced IRRs was 38.5% (5/13). The IRR group had a significantly higher BSA than the non-IRR group (median, 1.86 vs. 1.48 m^2^; *p* = 0.045). Additionally, rituximab dosage normalized by BSA in the IRR group was significantly lower than that in the non-IRR group (median, 268.8 vs. 337.9 mg/m^2^; *p* = 0.045).

**Conclusions:**

Our study revealed that adults with frequently relapsing MCNS who experienced IRRs tend to have a higher BSA, even with fixed-dose rituximab treatment. Therefore, when patients with higher BSA receive rituximab treatment, clinicians should be careful about monitoring patient condition whether the dosage is fixed or not.

## Background

Rituximab is an anti-cluster of differentiation 20 (CD20) monoclonal antibody widely used to treat various diseases, including hematologic malignancies, rheumatoid arthritis, and refractory nephrotic syndrome. Rituximab is a chimeric mouse-human monoclonal antibody, often causing infusion-related reactions (IRRs) [1]. Severe rituximab-induced IRRs have an incidence of approximately 10% [2] and require prompt medical intervention when they occur. Therefore, identifying patients at a high risk of rituximab-induced IRRs is clinically important.

Predictive markers of rituximab-induced IRRs have been explored. Factors strongly related to tumors, including tumor mass, bone marrow infiltration, high soluble interleukin-2 receptor levels, and low hemoglobin levels, have been reported as predictive markers [3–6]. In our previous study involving renal transplant recipients without malignancy, male sex, high body weight, body surface area (BSA), and body mass index (BMI) were found to be associated with rituximab-induced IRRs [7]. As rituximab dosage correlates with BSA, these four factors may be associated with rituximab-induced IRRs. However, as the rituximab dosage administered in the previous study was not fixed, evaluation focusing on physique-related factors has not been fully implemented. Therefore, whether higher dosage or physique-related factors are more strongly associated with rituximab-induced IRRs is unclear.

This study focused on patients who received fixed-dose rituximab treatment. In the Kidney Disease: Improving Global Outcomes guideline, rituximab is recommended for the treatment of adults with frequently relapsing minimal change nephrotic syndrome (MCNS) who previously received or wished to avoid cyclophosphamide [8]. In Japan, the dosage of rituximab for childhood-onset frequently relapsing MCNS is 375 mg/m^2^ with an upper limit of 500 mg [9], and the efficacy for adults has been reported [10]. We aimed to clarify the relationship between physique-related factors and rituximab-induced IRRs in adults with frequently relapsing MCNS who received 500 mg of rituximab.

## Materials and methods

### Patients and study design

This retrospective study included patients who received 500 mg of rituximab for frequently relapsing MCNS between September 2015 and November 2022 at the Department of Nephrology of the Osaka Metropolitan University Hospital. The inclusion criteria were (1) initial rituximab infusion; (2) premedication including 2 mg of oral or 5 mg of intravenous *d*-chlorpheniramine maleate and 200 mg or 400 mg of oral acetaminophen; (3) BSA ≥ 1.33m^2^; and (4) initial rituximab infusion at 25 mg/h. The exclusion criteria were (1) body temperature ≥ 37.0 °C immediately before rituximab infusion and (2) systolic blood pressure (sBP) < 90 mmHg immediately before rituximab infusion.

In our institution, *d*-chlorpheniramine maleate, acetaminophen, and 4 mg of intravenous betamethasone should be administered as premedication before rituximab administration to patients with frequently relapsing MCNS. However, when patients have received prednisolone for the treatment of frequently relapsing MCNS, physicians can omit betamethasone administration to avoid overdose of steroids.

For rituximab administration, 500 mg of rituximab was mixed with 450 mL of normal saline to a final concentration of 1 mg/mL. The mixed solution was initially administered at 25 mL/h, increased to 50 mL/h after 30 min, and increased to 100 and 200 mL/h every proceeding hour in the absence of adverse events, such as IRRs.

### Clinical data

Information regarding patient baseline characteristics, which are listed in Table 1, laboratory data (white blood cell count, hemoglobin level, platelet count, aspartate aminotransferase level, alanine transaminase level, serum creatinine level, estimated glomerular filtration rate, and serum albumin level), and vital signs (body temperature and sBP) were collected from medical records. “Regular use” was defined as use of drugs on the day of rituximab administration, except for *d*-chlorpheniramine maleate and acetaminophen premedication. When patients experienced IRRs, regular use was considered before the occurrence of IRRs. The prednisolone dosage was defined as the total amount of prednisolone administered on the day of rituximab administration.

**Table 1:** Patient baseline information and characteristics

Total patients		13
Sex	Male	7 (53.8%)
	Female	6 (46.2%)
Age (years)	Median [25, 75%]	28 [23–30]
Body weight (kg)	Median [25, 75%]	53.0 [47.4–70.2]
	Mean ± SD	59.1 ± 16.9
BSA (m^2^)	Median [25, 75%]	1.52 [1.47–1.74]
	Mean ± SD	1.62 ± 0.23
BMI (kg/m^2^)	Median [25, 75%]	19.0 [18.3–27.3]
	Mean ± SD	22.2 ± 5.1
Regular use of drugs	Histamine H1 blocker	0 (0.0%)
	Histamine H2 blocker	4 (30.8%)
	Analgesic drug	0 (0.0%)
	Prednisolone	13 (100%)
	Cyclosporine	10 (76.9%)
	Mizoribine	1 (7.7%)
	Mycophenolate mofetil	0 (0.0%)
Prednisolone dosage (mg/day)	Median [25, 75%]	17.5 [10.0–25.0]
Details of premedication (presence/absence of 4 mg of intravenous betamethasone)	Presence	11 (84.6%)
	Absence	2 (15.4%)

BSA: body surface area; BMI: body mass index; SD: standard deviation;

### Definition of IRRs

IRRs were defined as any of the following symptoms, occurring from the start of rituximab administration until 8:00 am on the following day, and recorded by physicians or nurses in patient charts: body temperature > 38.0 °C, chills, malaise, throat discomfort, itching, headache, facial flushing, hypotension, nausea, and vomiting. Hypotension was defined as the lowest measured sBP value below 90 mmHg or a 30% decrease in sBP compared with that before rituximab administration. These symptoms were graded according to the National Cancer Institute Common Terminology Criteria for Adverse Events, version 5.0.

### Statistical analysis

Categorical variables were analyzed using Fisher’s exact test and continuous variables were analyzed using the Mann–Whitney *U* test. Statistical significance was set at *p*-values of < 0.05. All statistical analyses were performed using EZR version 1.55 (Saitama Medical Center, Jichi Medical University, Saitama, Japan), a graphical user interface for R (The R Foundation for Statistical Computing, Vienna, Austria) [11].

## Results

### Patient characteristics

Thirteen patients received an initial dose of 500 mg of rituximab for frequently relapsing MCNS, and none were excluded from this study. All eligible patients were given prednisolone not as premedication but for frequently relapsing MCNS. Table 1 shows the baseline patient characteristics.

### Details of rituximab-induced IRRs

Five of 13 (38.5%) patients experienced IRRs; all IRRs were grade ≤ 2 (Table 2). Three patients experienced a single symptom—one patient each experienced throat discomfort, itching, and hypotension. The other patients experienced multiple symptoms—one of malaise and throat discomfort and one of hypotension and throat discomfort.

**Table 2:** Characterization of infusion-related reactions

Symptom	Grade	N
only itching	G2	1
only throat discomfort	G1	1
only hypotension	G1	1
malaise and throat discomfort	G1	1
hypotension and throat discomfort	G1	1

### Clinical characteristics of patients who experienced IRRs

Table 3 presents the analysis results of the clinical characteristics of patients who experienced IRRs. The IRR group had a significantly higher BSA than the non-IRR group (median, 1.86 vs. 1.48 m^2^; *p* = 0.045). Thus, rituximab dosage normalized by BSA in the IRR group was significantly lower than that in the non-IRR group (median, 268.8 vs. 337.9 mg/m^2^; *p* = 0.045). No other factors differed significantly between the groups.

**Table 3:** Comparison of clinical characteristics between the infusion-related reaction and non-infusion-related reaction groups

		IRR group (*n* = 5)	non-IRR group (*n* = 8)	*p*-Value
Patient characteristics				
Age (years)	Median [25, 75%]	29 [26–30]	26 [23–31]	1.0
Sex	Male	3 (60.0%)	4 (50.0%)	1.0 ^*a)*^
	Female	2 (40.0%)	4 (50.0%)	
Body weight (kg)	Median [25, 75%]	82.1 [53.0–85.5]	50.2 [46.1–54.4]	0.065
BSA (m^2^)	Median [25, 75%]	1.86 [1.58–2.02]	1.48 [1.44–1.55]	0.045
BMI (kg/m^2^)	Median [25, 75%]	27.7 [19.0–29.2]	18.5 [17.8–22.8]	0.093
Laboratory data				
White blood cell count (*10^2^/µL)	Median [25, 75%]	100 [98–114]	68 [61–88]	0.067
Hemoglobin value (g/dL)	Median [25, 75%]	13.4 [12.0–15.8]	14.3 [13.2–14.4]	0.94
Platelet count (*10^4^/µL)	Median [25, 75%]	31.4 [28.7–44.5]	26.6 [22.7–32.0]	0.28
Aspartate aminotransferase (U/L)	Median [25, 75%]	18 [17–18]	18 [15–21]	0.82
Alanine transaminase (U/L)	Median [25, 75%]	16 [9–26]	24 [19–38]	0.13
Serum creatinine (mg/dL)	Median [25, 75%]	0.73 [0.56–0.84]	0.72 [0.66–0.79]	0.88
eGFR (mL/min/1.73 m^2^)	Median [25, 75%]	103.9 [89.3–107.5]	92.7 [75.4–102.0]	0.52
Serum albumin (g/dL)	Median [25, 75%]	4.1 [2.9–4.1]	3.6 [3.3–3.8]	0.51
Regular use of drugs on the day of rituximab				
Histamine H1 blocker	Yes	0 (0.0%)	0 (0.0%)	1.0 ^*a)*^
	No	5 (100%)	8 (100%)	
Histamine H2 blocker	Yes	2 (40.0%)	2 (25.0%)	1.0 ^*a)*^
	No	3 (60.0%)	6 (75.0%)	
Analgesic drug	Yes	0 (0.0%)	0 (0.0%)	1.0 ^*a)*^
	No	5 (100%)	8 (100%)	
Prednisolone	Yes	5 (100%)	8 (100%)	1.0 ^*a)*^
	No	0 (0.0%)	0 (0.0%)	
Cyclosporine	Yes	4 (80.0%)	6 (75.0%)	1.0 ^*a)*^
	No	1 (20.0%)	2 (25.0%)	
Mizoribine	Yes	0 (0.0%)	1 (12.5%)	1.0 ^*a)*^
	No	5 (100%)	7 (87.5%)	
Mycophenolate mofetil	Yes	0 (0.0%)	0 (0.0%)	1.0 ^*a)*^
	No	5 (100%)	8 (100%)	
Prednisolone dosage (mg/day)	Median [25, 75%]	12.5 [10–30]	18.8 [13.1–25.0]	1.0
Details of premedication (presence/absence of 4 mg of intravenous betamethasone)				
	Presence	4 (80.0%)	7 (87.5%)	1.0 ^*a)*^
	Absence	1 (20.0%)	1 (12.5%)	
Rituximab dosage normalized by BSA				
Dose/BSA (mg/m^2^)	Median [25, 75%]	268.8 [247.5–316.5]	337.9 [322.9–346.8]	0.045

*a)* Fisher’s exact test. Other: Mann–Whitney *U* test

IRRs: infusion-related reactions; BSA: body surface area; BMI: body mass index; eGFR: estimated glomerular filtration rate

## Discussion

This retrospective study was conducted to clarify the relationship between physique-related factors and rituximab-induced IRRs in adults with frequently relapsing MCNS who received 500 mg of rituximab. Our study revealed that, even with fixed-dose rituximab treatment, the IRR group had a significantly higher BSA than the non-IRR group. Owing to the fixed rituximab dosage in this study, the rituximab dosage normalized by BSA in the IRR group was lower than that in the non-IRR group.

In a previous study involving adults with nephrotic syndrome, the incidence of IRRs was 46.3% (25/54) at the first infusion of rituximab [12], whereas that in the present study was 38.5% (5/13), indicating only a minor difference. The data of BSA in the previous study were not shown; however, the body weight and BMI of eligible patients in both studies were similar. Therefore, we believe that the BSA of patients enrolled in both studies was also similar. Hence, the similarity between these rates likely results from the similarity in BSA.

In this study, we revealed that the BSA in the IRR group was significantly higher than that in the non-IRR group in adults with frequently relapsing MCNS, consistent with our previous findings [7]. There is a positive correlation between BSA and blood volume [13]. Therefore, patients with higher BSA may have more peripheral B cells owing to the increase in blood volume. With ocrelizumab, another anti-CD20 antibody, an increase in the number of peripheral B cells and their rapid destruction are linked to the incidence of IRRs [14]. As rituximab is also an anti-CD20 antibody, an increase in peripheral B-cell count may be associated with IRRs, not only with fixed-dose ocrelizumab but also with fixed-dose rituximab. Hence, BSA may be more strongly associated with rituximab-induced IRRs than with high rituximab dosages.

In our previous study, male sex, high body weight, and high BMI were also found to be predictive markers for rituximab-induced IRRs [7]. However, the proportion of patients who were male and had a high body weight and BMI in the IRR group tended to be higher than that in the non-IRR group. This statistical discrepancy may be due to the small number of eligible patients.

In a previous study on ocrelizumab, a high BMI was identified as a predictive marker for IRRs, although the dosage was fixed [14]. The authors hypothesized that the inflammatory responses caused by adipose tissues result in increased peripheral B-cell count in obese patients [14]. Similarly, Gregor et al. reported the increased secretion of many inflammatory cytokines in obese tissue [15]. Moreover, another previous study reported an increase in the percentage of B cells in the lymphocyte compartment in obese patients [16]. Therefore, BMI, an index of obesity, may also be associated with the increase in the number of peripheral B cells. In this study, although there was no significant difference, the BMI in the IRR group tended to be higher than that in the non-IRR group. Additionally, as the median BMI of patients who experienced IRRs in this study was ≥ 25 kg/m^2^, the majority met the definition of obesity in Japan. Hence, an increasing number of peripheral B cells due to obesity might also be associated with IRRs for rituximab.

This study has some limitations. First, this was a single-institution retrospective study, and the sample size was small. Therefore, we could not perform a multivariate analysis, adjust the *p*-value to eliminate the possibility of type one error, and calculate a meaningful cutoff for BSA. However, the main outcome of our study was the examination of the relationship between physique-related factors and rituximab-induced IRRs, and our results were consistent with those of previous studies [5, 7]. Consequently, we considered that our results were reliable, and the possibility of type one error was low. In the future, studies including a larger number of patients are needed to confirm the validity of our findings and obtain a meaningful cutoff for BSA to predict rituximab-induced IRRs. Second, in this study, B-cell counts were not routinely measured. Therefore, whether peripheral B-cell count increases in patients with higher BSA is unclear. Additionally, considering the characteristics of nephrotic syndrome, body weight gain due to edema might affect BSA accuracy because it was calculated by the Dubois and Dubois formula. Therefore, further studies, including measurement of B-cell counts, are needed to clarify the relationship between BSA and rituximab-induced IRRs. Third, we could not collect information on adipose tissue as an important factor for increasing the peripheral B-cell count. Therefore, the extent of the association of rituximab-induced IRRs with adipose tissue is still unclear. Further studies, including an evaluation of adipose tissues, are needed to clarify the relationship between physique-related factors and rituximab-induced IRRs.

## Conclusions

Our study revealed that adults with frequently relapsing MCNS who experienced IRRs tend to have a higher BSA, even with fixed-dose rituximab treatment. Therefore, when patients with higher BSA receive rituximab treatment, clinicians should be careful about patient condition whether the dosage is fixed or not.

## Data Availability

Due to the nature of this research, the participants did not agree for their data to be shared publicly; therefore, supporting data are not available.

## Abbreviations

IRRs: Infusion-related reactions
BSA: Body surface area
MCNS: Minimal change nephrotic syndrome
CD20: Cluster of differentiation 20
eGFR: Estimated glomerular filtration rate
BMI: Body mass index
sBP: Systolic blood pressure
